# Epidemiological characteristics and transmissibility of shigellosis in Hubei Province, China, 2005 – 2017

**DOI:** 10.1186/s12879-020-04976-x

**Published:** 2020-04-07

**Authors:** Qi Chen, Jia Rui, Qingqing Hu, Ying Peng, Hao Zhang, Zeyu Zhao, Yeqing Tong, Yang Wu, Yanhua Su, Benhua Zhao, Xuhua Guan, Tianmu Chen

**Affiliations:** 1 Hubei Provincial Center for Disease Control and Prevention, NO.6 Zhuodaoquan North Road, Hongshan District, Wuhan City, Hubei Province People’s Republic of China; 2 State Key Laboratory of Molecular Vaccinology and Molecular Diagnostics, School of Public Health, Xiamen University, 4221-117 South Xiang’an Road, Xiang’an District, Xiamen City, Fujian Province People’s Republic of China; 3 Division of Public Health, School of Medicine, University of Utah, 201 Presidents Circle, Salt Lake City, UT 84112 USA; 4 Wuhan Center for Disease Control and Prevention, Wuhan City, Hubei Province People’s Republic of China; 5 Yichang Center for Disease Control and Prevention, Yichang City, Hubei Province People’s Republic of China

**Keywords:** Shigellosis, Transmissibility, Effective reproduction number, Mathematical model

## Abstract

**Background:**

Shigellosis is one of the main diarrhea diseases in developing countries. However, the transmissibility of shigellosis remains unclear.

**Methods:**

We used the dataset of shigellosis cases reported between January 2005 and December 2017, from Hubei Province, China. A mathematical model was developed based on the natural history and the transmission mechanism of the disease. By fitting the data using the model, transmission relative rate from person to person (*b*) and from reservoir to person (*b*_*w*_), and the effective reproduction number (*R*_*eff*_) were estimated. To simulate the contribution of *b* and *b*_*w*_ during the transmission, we performed a “knock-out” simulation in four scenarios: A) *b* = 0 and *b*_*w*_ = 0; B) *b* = 0; C) *b*_*w*_ = 0; D) control (no intervention).

**Results:**

A total of 130,770 shigellosis cases were reported in Hubei province, among which 13 cases were dead. The median annual incidence was 19.96 per 100,000 persons (range: 5.99 per 100,000 persons – 29.47 per 100,000 persons) with a decreased trend (trend *χ*^2^ = 25,470.27, *P* < 0.001). The mean values of *b* and *b*_*w*_ were 0.0898 (95% confidence interval [CI]: 0.0851–0.0946) and 1.1264 × 10^− 9^ (95% CI: 4.1123 × 10^− 10^–1.8416 × 10^− 9^), respectively. The “knock-out” simulation showed that the number of cases simulated by scenario A was almost the same as scenario B, and scenario C was almost the same as scenario D. The mean value of *R*_*eff*_ of shigellosis was 1.19 (95% CI: 1.13–1.25) and decreased slightly with a Linear model until it decreased to an epidemic threshold of 0.99 (95% CI: 0.65–1.34) in 2029.

**Conclusions:**

The incidence of shigellosis is still in high level. The transmissibility of the disease is low in Hubei Province. The transmission would be interrupted in the year of 2029.

## Background

Shigellosis, caused by *Shigella spp* which are Gram-negative bacteria, is a fecal-oral transmission disease with a symptom of severe colitis and induced by a very low infectious dose [1, 2]. In developing countries, *Shigella spp* are one of the top four attributable causes of moderate–severe diarrhea in children younger than 5 years. Shigellosis is an illness that kills roughly 750,000 people per year [3–5]. Although the incidence of shigellosis has declined remarkably due to the rapid improvements in water supply and sanitation in China [6], a considerable disease burden still exists and is unevenly distributed across the country [7–9]. Because of the different transmission conditions resulted from social-economic factors such as water supply and hygiene practices in different areas, the transmission of shigellosis is heterogeneous and is still a major public health problem in many areas in China [7, 10, 11]. The transmission interruption has a public importance in China as well as many developing countries. Generally, whether an infectious disease spreads or not depends on the size of transmissibility or “transmission force” behind the transmission. However, to our knowledge, there is no study investigating the transmissibility of shigellosis. Therefore, an understanding the transmissibility of shigellosis is of great significance to ensure a better disease prevention and control.

The transmissibility of an infectious disease is commonly quantified by basic reproduction number (*R*_0_), which is defined as the expected number of secondary infections that result from introducing a single infected individual into an otherwise susceptible population [12–15]. However, because of the disease intervention or the decreasing proportion of susceptible individuals due to the spread of the pathogen, it is hard to estimate *R*_0_. Therefore, effective reproduction number (*R*_*eff*_) is commonly employed instead, with the definition of the number of new cases which was produced by a typical case during the period of infection [16, 17]. From the definition, it is clear that when *R*_*eff*_ > 1, the disease is able to spread in the population. If *R*_*eff*_ < 1, the infection will be cleared from the population. Therefore, *R*_*eff*_ = 1 is considered as the “epidemic threshold”.

Although statistical model, like Bayesian model, was employed to explore the epidemiological characteristics [18], there is few research to simulate and forecast the population-based transmission [19]. To explore the transmissibility, we have performed a study to estimate the relative transmissibility of shigellosis among male and female individuals in Hubei Province, China [20]. And we found that the transmissibility of shigellosis was different in females was higher than that of males [20]. However, we still do not know the values of *R*_*eff*_ and their trends. In this study, we used the dataset of reported shigellosis cases collected in Hubei Province, China, developed a mathematical model to fit the data, estimated the values and trends of *R*_*eff*_, and forecasted the transmission interruption year of the disease.

## Methods

### Data collection

Hubei Province, located in the center of China and north of the Dongting Lake, has a population of more than 58 million. Hubei is known as the “province of thousands of lakes”. Except the main stream of the Yangtze River and Han River, there are 4228 rivers and 755 lakes in the province. The large water areas provide an easy spread of *Shigella spp*. Therefore, it is of great value to study shigellosis transmissibility in Hubei Province, China.

In this study, we used the dataset of reported shigellosis cases that was built from January 2005 to December 2017 in the province. The sex, age, illness onset date, and diagnosis date of each case was included in the data. Incidences of 12 urban areas (including 7 central districts in Wuhan City and 5 districts in Yichang City) and 14 rural areas (including 5 outer suburbs districts in Wuhan City, and 8 counties or county-level cities in Yichang City) from 2005 to 2017 were collected to compare the difference between urban and rural areas. All the cases were collected from the China Information System for Disease Control and Prevention. All the cases were identified following the case definitions from “Diagnostic criteria for bacterial and amoebic dysentery (WS287-2008)” announced by the National Health Commission of the People’s Republic of China:

A) Suspected cases: Diarrhea, with purulent or mucous stool or watery stool or sparse stool, accompanied by severe symptoms after internal emergencies, has not yet been identified form other causes of diarrhea.
B) Clinically diagnosed cases: A patient who has the following 4 items: 1) unclean diet and/or contact history with dysentery patients; 2) sudden onset, chills and high fever, followed by abdominal pain, diarrhea and internal urgency, severe defecation 10 to 20 times a day, but not much, purulent stool, and moderate systemic poisoning symptoms; or severe symptoms like convulsions, headache, systemic muscle ache, dehydration and electrolyte disorders, may have left lower abdominal tenderness with bowel ringing hyperactivity; 3) routine stool examination showed that white blood cells or pus cells > = 15/HPF (400 folds), erythrocyte and phagocyte could be seen; 4) excluding diarrhea caused by other causes.
C) Confirmed cases: *Shigella spp* was tested positive by fecal culture from clinical diagnosis of cases.

In this study, we only included clinically diagnosed cases and confirmed cases for the analysis.

### The transmission models

In this study, two models were developed according to the transmission routes of the disease. According to our previous research [14, 19], we firstly built a susceptible-exposed-symptomatic-asymptomatic-recovered-water/food (SEIARW) model (denoted as Model 1) in which two routes (person–to–person and reservoir–to–person) were considered. For the route of person–to–person, two routes (symptomatic–to–susceptible and asymptomatic–to–susceptible) were both considered. In the model, people were divided into susceptible (*S*), exposed (*E*), symptomatic (*I*), asymptomatic (*A*), and recovered (*R*) individuals. The reservoir, including water or food, was denoted as *W*. In order to normalize the dimensions of people and *Shigella spp* population, we set *s* = *S*/*N*, *e* = *E*/*N*, *i* = *I*/*N*, *a* = *I*/*A*, *r* = *R*/*N*, *w* = *εW*/*μN*, *b* = *βN*, and *b*_*w*_ =* μβ*_*W*_*N*/*ε*.

In the model, *N* is assumed to denote the total population. The parameter *β* is the transmission relative rate from person to person, *β*_*W*_ is the transmission relative rate from reservoir to person, *k* is the relative transmissibility of asymptomatic to symptomatic individuals, *ω* is the incubation relative rate, *p* is the proportion of asymptomatic individuals, *γ* is the infectious period relative rate of symptomatic individuals, *γ’* is the infectious period relative rate of asymptomatic individuals, *ε* is the relative rate of the pathogen’s lifetime, *c* is the shedding rate of the asymptomatic comparing to the infectious, and *μ* is the pathogen shedding coefficient of infectious individuals. The equations of the model are as follows:

$$ \frac{ds}{dt}=- bs\left(i+ ka\right)-{b}_w sw $$

$$ \frac{de}{dt}= bs\left(i+ ka\right)+{b}_w sw-\omega e $$

$$ \frac{di}{dt}=\left(1-p\right) we-\gamma i $$

$$ \frac{da}{dt}= pwe-{\gamma}^{\prime }a $$

$$ \frac{dr}{dt}=\gamma i+{\gamma}^{\prime }a $$

$$ \frac{dw}{dt}=\varepsilon \left(i+ ca-w\right) $$

We also developed a susceptible-exposed-symptomatic-asymptomatic-recovered (SEIAR) model (denoted as Model 2) which only includes the transmission route of person–to–person [21–23]. The equations of the model are as follows:

$$ \frac{ds}{dt}=- bs\left(i+ ka\right) $$

$$ \frac{de}{dt}= bs\left(i+\mathrm{k}a\right)-\omega e $$

$$ \frac{di}{dt}=\left(1-p\right) we-\gamma i $$

$$ \frac{da}{dt}= pwe-{\gamma}^{\prime }a $$

$$ \frac{dr}{dt}=\gamma i+{\gamma}^{\prime }a $$

In the model, the effective reproduction number (*R*_*eff*_) was calculated by the equation as follows:

$$ {R}_{eff}= bs\left(\frac{1-p}{\gamma }+\frac{kp}{\gamma^{\prime }}\right) $$

### Parameter estimation

There are eight parameters (*b*, *b*_*w*_, *k*, *ω*, *p*, *γ*, *γ’*, *c*, and *ε*) in the above models. According to our previous research [19], *k*, *ω*, *p*, *γ*, *γ’*, *c*, and *ε* are disease-specific parameters which could be estimated from literatures. The incubation period of shigellosis is 1–4 days [24, 25], and most commonly 1 day, therefore, *ω* = 1.0. The proportion of asymptomatic infection ranges from 0.0037 to 0.27 [26–28], and it can be set *p* = 0.1. The infectious period of symptomatic infection is 13.5 days [19], therefore, *γ* = 0.0741. According to our previous research [19], the infectious period of asymptomatic infection could be simulated 5 weeks in our model, thus *γ’* = 0.0286. Because of that the die-off rate of *Shigella spp* is about 24.5 h [29], *ε* = 0.6931 was consequently simulated [19]. In our previous research [19], a typical case has diarrhea about 3.2 times (range 3–12 times) per day but an asymptomatic individual only sheds stool once per day, thus *c* = 0.3125. Due to reduction of shedding frequency, the relative transmissibility of asymptomatic individual (*k*) was modeled to be a reduced quantity as 0.3125 [19].

However, *b* and *b*_*w*_ are scenario- or area-specific parameters which depend on different outbreaks and periods. Therefore, these two parameters are confirmed by curve fitting by Models 1 and 2 to the collected data. To simulate the contribution of *b* and *b*_*w*_ during the transmission, we performed a “knock-out” simulation which was based on the following four scenarios: A) *b* = 0 and *b*_*w*_ = 0; B) *b* = 0; C) *b*_*w*_ = 0; D) control (no intervention).

### Simulation method and statistical analysis

Berkeley Madonna 8.3.18 (developed by Robert Macey and George Oster of the University of California at Berkeley. Copyright©1993–2001 Robert I. Macey & George F. Oster) was employed for model simulation and least root mean square was adopted to assess the goodness of fit. The simulation methods were the same as the previously publications [12, 14, 19, 30, 31]. The chi-square and trend chi-square tests and *t* test were performed by SPSS 13.0 (IBM Corp., Armonk, NY, USA).

Eleven equations (Linear, Logarithmic, Inverse, Quadratic, Cubic, Compound, Power, S, Growth, Exponential, Logistic) were employed to fit the data of *R*_*eff*_ which was calculated by the reported data. The equations of the 11 models were shown as follows:

$$ \mathrm{Linear}:f(x)={b}_0+{b}_1x $$

$$ \mathrm{Logarithmic}:f(x)={b}_0+{b}_1\ln (x) $$

$$ \mathrm{Inverse}:f(x)={b}_0+\frac{b_1}{x} $$

$$ \mathrm{Quadratic}:f(x)={b}_0+{b}_1x+{b}_2{x}^2 $$

$$ \mathrm{Cubic}:f(x)={b}_0+{b}_1x+{b}_2{x}^2+{b}_3{x}^3 $$

$$ \mathrm{Compound}:f(x)={b}_0+{b}_1^x $$

$$ \mathrm{Power}:f(x)={b}_0+{x}^{b_1} $$

$$ \mathrm{S}:f(x)={e}^{\left({b}_0+\frac{b_1}{x}\right)} $$

$$ \mathrm{Growth}:f(x)={e}^{\left({b}_0+{b}_1x\right)} $$

$$ \mathrm{Exponential}:f(x)={b}_0{e}^{b_1x} $$

$$ \mathrm{Logistic}:f(x)=\frac{1}{\frac{1}{u}+{b}_0+{b}_1^x} $$

In the equations, *x* and *f*(*x*) refer to time (year) and dependent variables (*R*_*eff*_), respectively; *b*_0_, *b*_1_, *b*_2_, *b*_3_, and *u* refer to the coefficients of the models which were estimated by curve fitting with the data. Determination coefficient (*R*^2^) was employed to evaluate the curve fitting.

## Results

### Reported shigellosis cases in Hubei Province

**Fig. 1:**
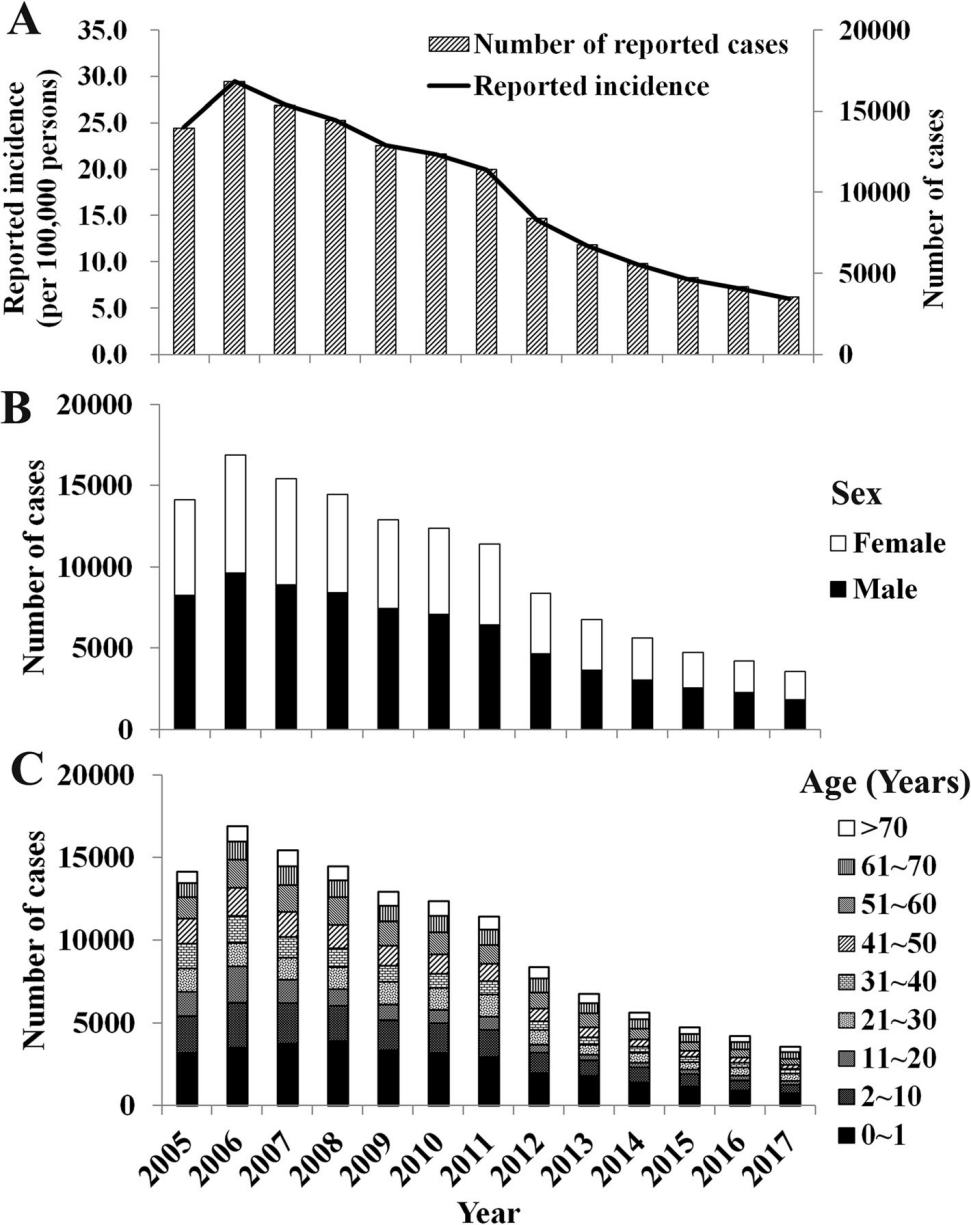
Epidemiological characteristics of Shigellosis cases from 2005 to 2017 in Hubei Province, China. **a**, reported cases and incidence of Shigellosis cases; **b**, sex distribution; **c**, age distribution

From January 1, 2005 to December 31, 2017, 130,770 Shigellosis cases were reported in the province. Among them 13 cases were dead with a case fatality rate of 0.01%. The median yearly reported incidence was 19.96 per 100,000 persons (range: 5.99 per 100,000 persons – 29.47 per 100,000 persons). Figure 1a showed that the number of reported cases and reported incidence yearly were both decreased significantly (trend *χ*^2^ = 25,470.27, *P* < 0.001).

**Fig. 2:**
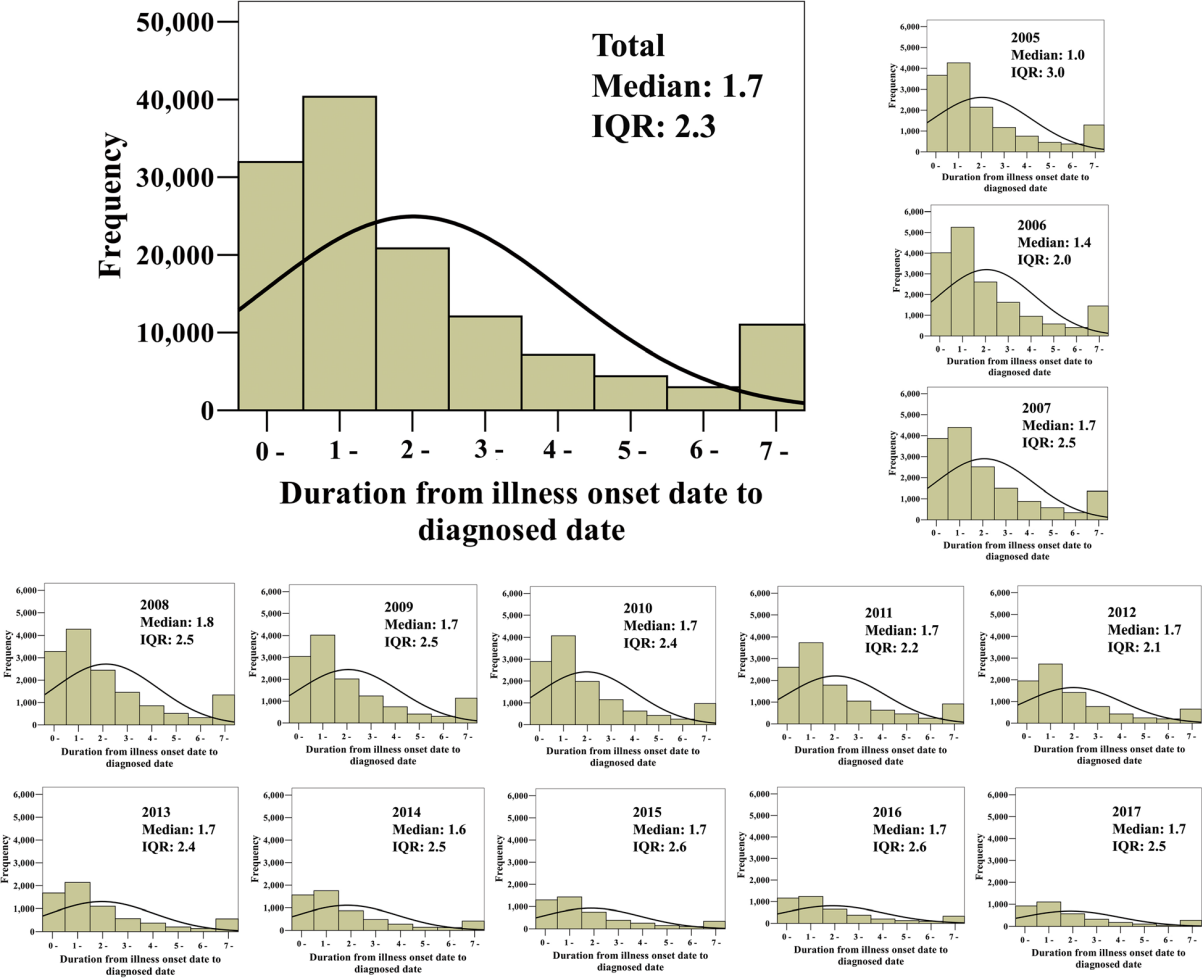
Distribution of duration from illness onset date to diagnosed date of 130,770 Shigellosis cases in Hubei Province, China. IQR, interquartile range

Of all the cases, male cases were account for 56.57% (73,981/130770) which was significantly higher than that of female cases (*χ*^2^ = 2255.19, *P* < 0.001). This incidence difference between male and female was observed in each year (Fig. 1b). Children cases with an age of 10 years and younger had the highest case proportion of 39.30%, with the “0 – 1” years old children or new-born cases were account for 61.33% (Table 1). The second age group with highest case proportion was “11 – 20” years (15.20%) followed by “61 –” years (14.51%). The distribution of incidence of age group was similar in different years (Fig. 1c). The median duration from illness onset date to diagnosed date (*D*_*ID*_) of all the cases was 1.7 days (inter-quartile range [IQR]: 2.3 days). Each year had a similar distribution of *D*_*ID*_ (Fig. 2). The *D*_*ID*_ of 71.24% cases were lower than 4 days, especially lower 1 days (24.43%) and 2 days (30.87%).

**Table 1:** Epidemiological characteristics of 130,770 reported Shigellosis cases in Hubei Province, China

Variables	Number of cases	Percentage (%)
Sex	130,770	100.00
Male	73,981	56.57
Female	56,789	43.43
Age (Years)	130,770	100.00
0~1	31,521	24.10
2~10	19,876	15.20
11~20	10,546	8.06
21~30	13,027	9.96
31~40	10,308	7.88
41~50	12,460	9.53
51~60	14,058	10.75
61~70	10,237	7.83
> 70	8737	6.68
*D*_*ID*_ (days)	130,770	100.00
0~	31,947	24.43
1~	40,367	30.87
2~	20,849	15.94
3~	12,072	9.23
4~	7147	5.47
5~	4392	3.36
6~	2981	2.28
7~	11,015	8.42

*D*_*ID*_, duration from illness onset date to diagnosed date

Among the 17 sub-regions of Hubei Province, Wuhan City had the most reported cases (39.88%) and the highest cumulative incidence during the past 13 years. The second highest number of cases was reported in Yichang City (8.66%) and Jingzhou City (8.24%). However, the rank of reported incidence was very different. Xiantao City and Yichang City had the second and third highest incidence, respectively. Shennongjia, which is a forest region, had the least reported cases (0.05%) and the lowest reported incidence (Table 2).

**Table 2:** Regional distribution of reported Shigellosis cases in Hubei Province, China, 2005–2017

City	Reported cases		Reported death cases	
	Number	Cumulative incidence (per 100,000)	Number	Case fatality rate (%)
Wuhan	51,948	482.51	3	0.01
Yichang	11,284	273.22	0	0.00
Jingzhou	10,739	188.47	0	0.00
Huanggang	7426	117.48	0	0.00
Enshi	7393	221.86	2	0.03
Xianning	6222	246.32	2	0.03
Xiaogan	5495	112.05	1	0.02
Xiangyang	4875	86.45	1	0.02
Xiantao	4494	388.48	0	0.00
Huangshi	4253	172.50	2	0.05
Shiyan	3851	112.96	0	0.00
Jingmen	3516	121.19	1	0.03
Tianmen	2980	220.07	1	0.03
Suizhou	2477	112.50	0	0.00
Qianjiang	2069	230.04	0	0.00
Ezhou	1172	109.69	0	0.00
Shennongjia	65	84.50	0	0.00

**Fig. 3:**
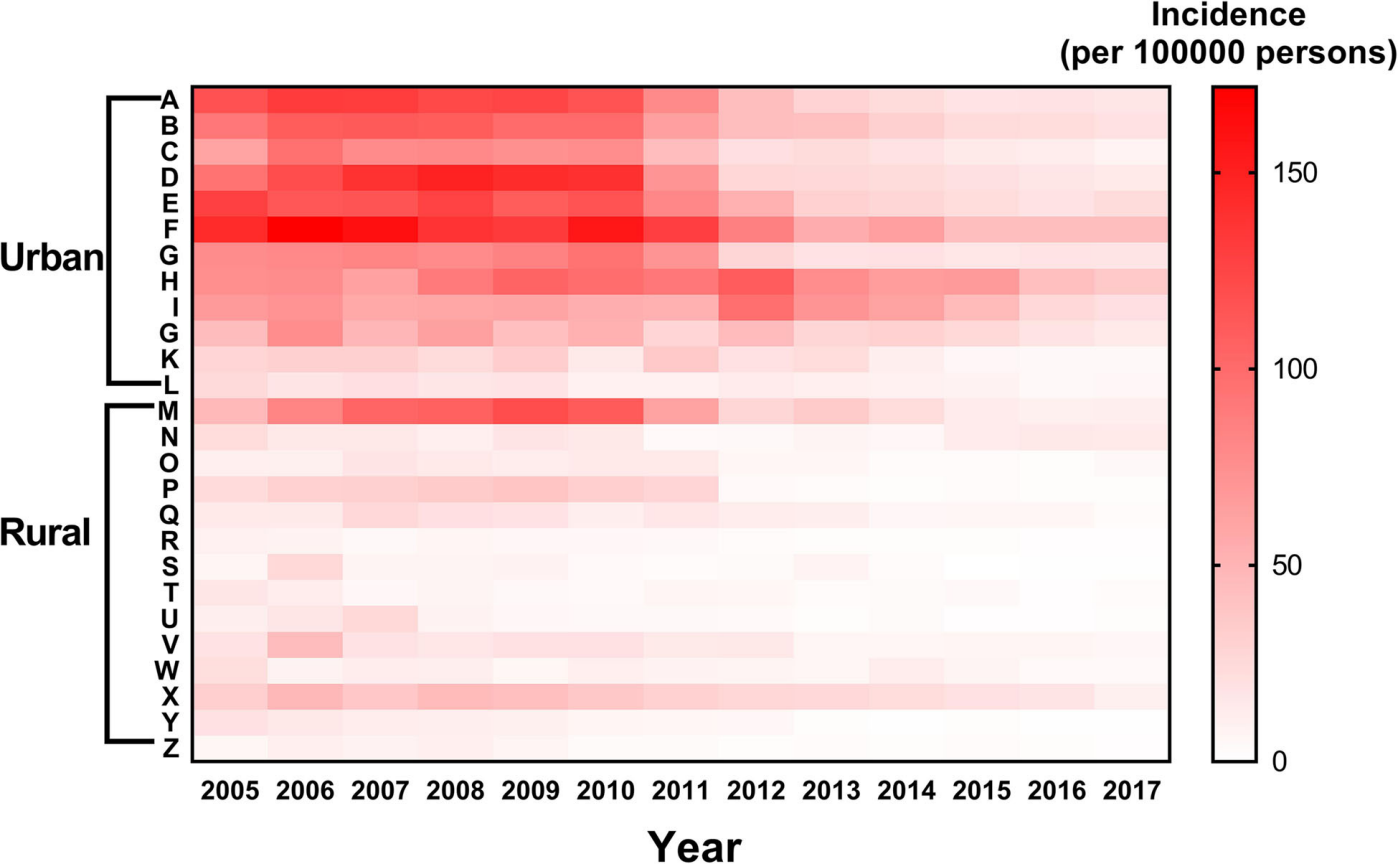
Reported incidences in 12 urban areas and 14 rural areas from 2005 to 2017 in Hubei Province, China

The mean reported incidence of shigellosis was 58.53 per 100,000 in urban areas (95%CI: 51.91 per 100,000–65.15 per 100,000) and 14.10 per 100,000 in rural areas (95% CI: 11.34 per 100,000–16.86 per 100,000) (Fig. 3). The difference of the incidences was statistically significant between urban and rural areas (*t* = 12.1345, *P* < 0.001).

### Curve fitting and the transmissibility of the disease

**Fig. 4:**
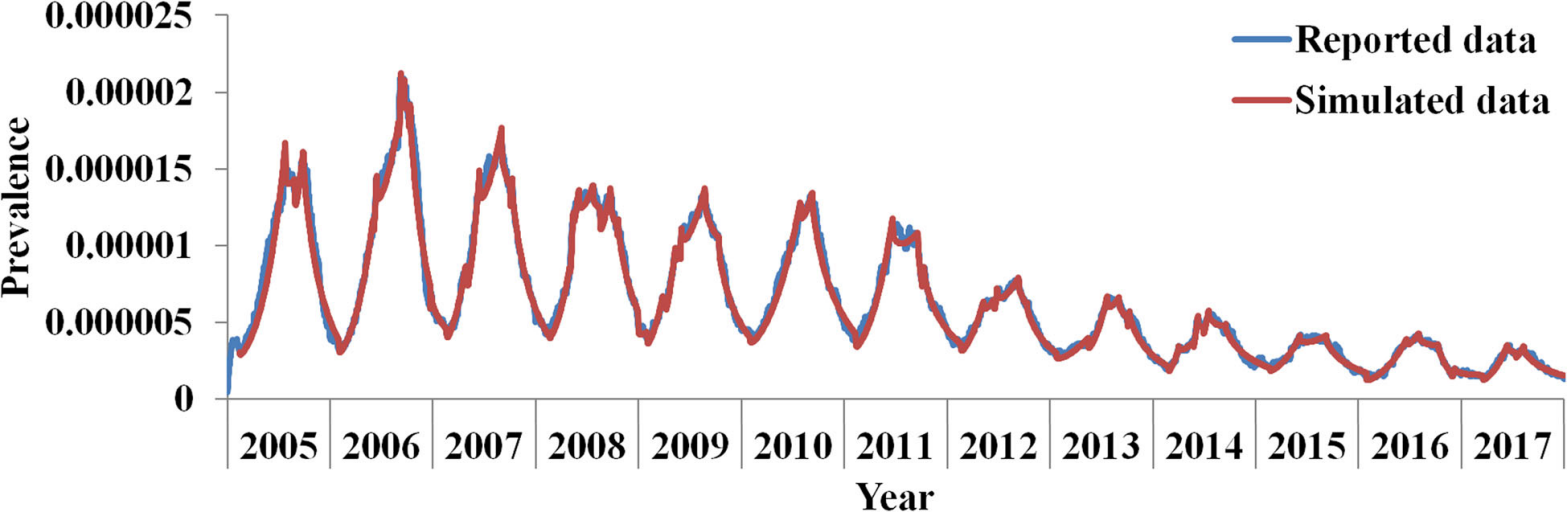
Curve fitting of Model 1 to reported data from 2005 to 2017 in Hubei Province, China

**Fig. 5:**
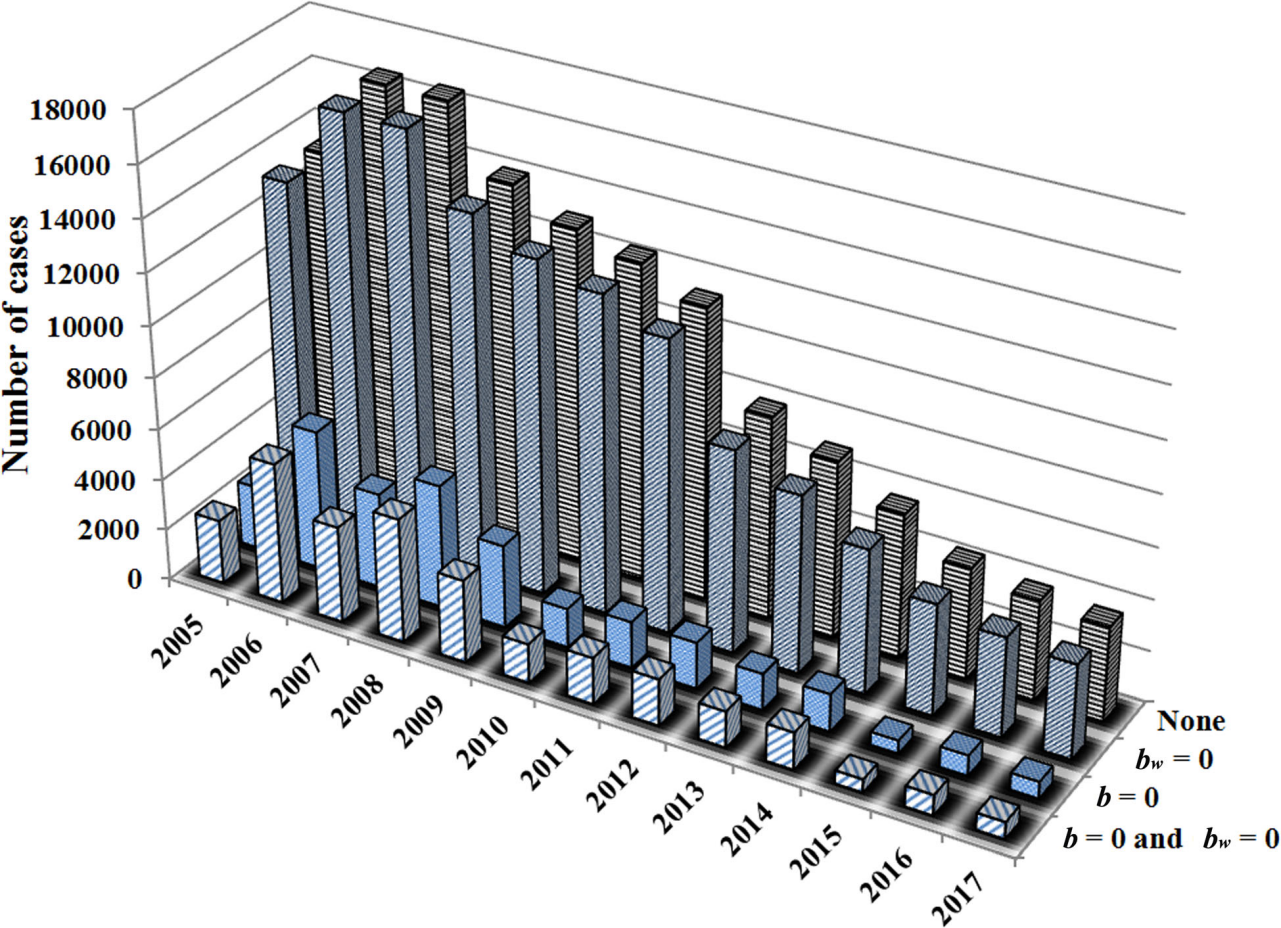
Four scenarios to simulate the contribution of *b* and *b*_*w*_ during the transmission (*b* = 0 and *b*_*w*_ = 0; *b* = 0; *b*_*w*_ = 0; and control denoted as “None”)

The results of curve fitting (Fig. 4) showed that Model 1 fitted well to the data (*χ*^2^ = 0.00015, *P* > 0.999). Calculated by the model, the mean value of *b* was 0.0898 (95% CI: 0.0851–0.0946) and *b*_*w*_ was 1.1264 × 10^− 9^ (95% CI: 4.1123 × 10^− 10^–1.8416 × 10^− 9^) from 2005 to 2017 in the province. The difference between the two parameters was as much as more than seven orders of magnitude in each year (Table 3). Results of the “knock-out” simulation (Fig. 5) showed that the number of cases simulated by scenario A (*b* = 0 and *b*_*w*_ = 0) was almost the same as that simulated by scenario B (*b* = 0), and that the number of cases simulated by scenario C (*b*_*w*_ = 0) was the almost same as that simulated by scenario D (control), which means that parameter *b*_*w*_ had almost no contribution to the transmission.

**Table 3:** Estimated values of *b* and *b*_*w*_ from 2005 to 2017 in Hubei Province, China

Year	*b*	95% CI of *b*		*b*_*w*_	95% CI of *b*_*w*_	
	Mean	Lower bound	Upper bound	Mean	Lower bound	Upper bound
2005	0.0816	0.0623	0.1009	8.5900 × 10^−9^	−4.0432 × 10^−9^	2.1223 × 10^−8^
2006	0.1054	0.0857	0.1250	5.5557 × 10^− 9^	2.8943 × 10^− 9^	8.2171 × 10^− 9^
2007	0.0881	0.0708	0.1055	1.9438 × 10^−11^	1.5076 × 10^−11^	2.3799 × 10^− 11^1
2008	0.0937	0.0753	0.1121	3.5762 × 10^−12^	1.7830 × 10^−12^	5.3694 × 10^−12^
2009	0.0917	0.0724	0.1109	3.4175 × 10^−13^	2.9759 × 10^−13^	3.8591 × 10^−13^
2010	0.0792	0.0569	0.1014	3.3700 × 10^− 13^	1.5747 × 10^− 13^	5.1653 × 10^− 13^
2011	0.0840	0.0561	0.1120	2.6340 × 10^− 13^	8.0393 × 10^−14^	4.4641 × 10^− 13^
2012	0.0845	0.0697	0.0993	1.4250 × 10^− 13^	1.1837 × 10^− 13^	1.6663 × 10^− 13^
2013	0.0908	0.0619	0.1197	9.2350 × 10^− 14^	5.0479 × 10^− 14^	1.3422 × 10^− 13^
2014	0.0905	0.0739	0.1072	1.2351 × 10^− 14^	5.5676 × 10^−15^	1.9135 × 10^− 14^
2015	0.0787	0.0590	0.0983	4.6233 × 10^−15^	9.4468 × 10^−16^	8.3020 × 10^− 15^
2016	0.0889	0.0556	0.1221	3.1740 × 10^− 15^	2.5692 × 10^− 15^	3.7788 × 10^− 15^
2017	0.0816	0.0697	0.0935	2.7180 × 10^− 15^	1.4383 × 10^− 15^	3.9977 × 10^− 15^
Pooled	0.0898	0.0851	0.0946	1.1264 × 10^− 9^	4.1123 × 10^−10^	1.8416 × 10^− 9^

*CI* Confidence interval

**Fig. 6:**
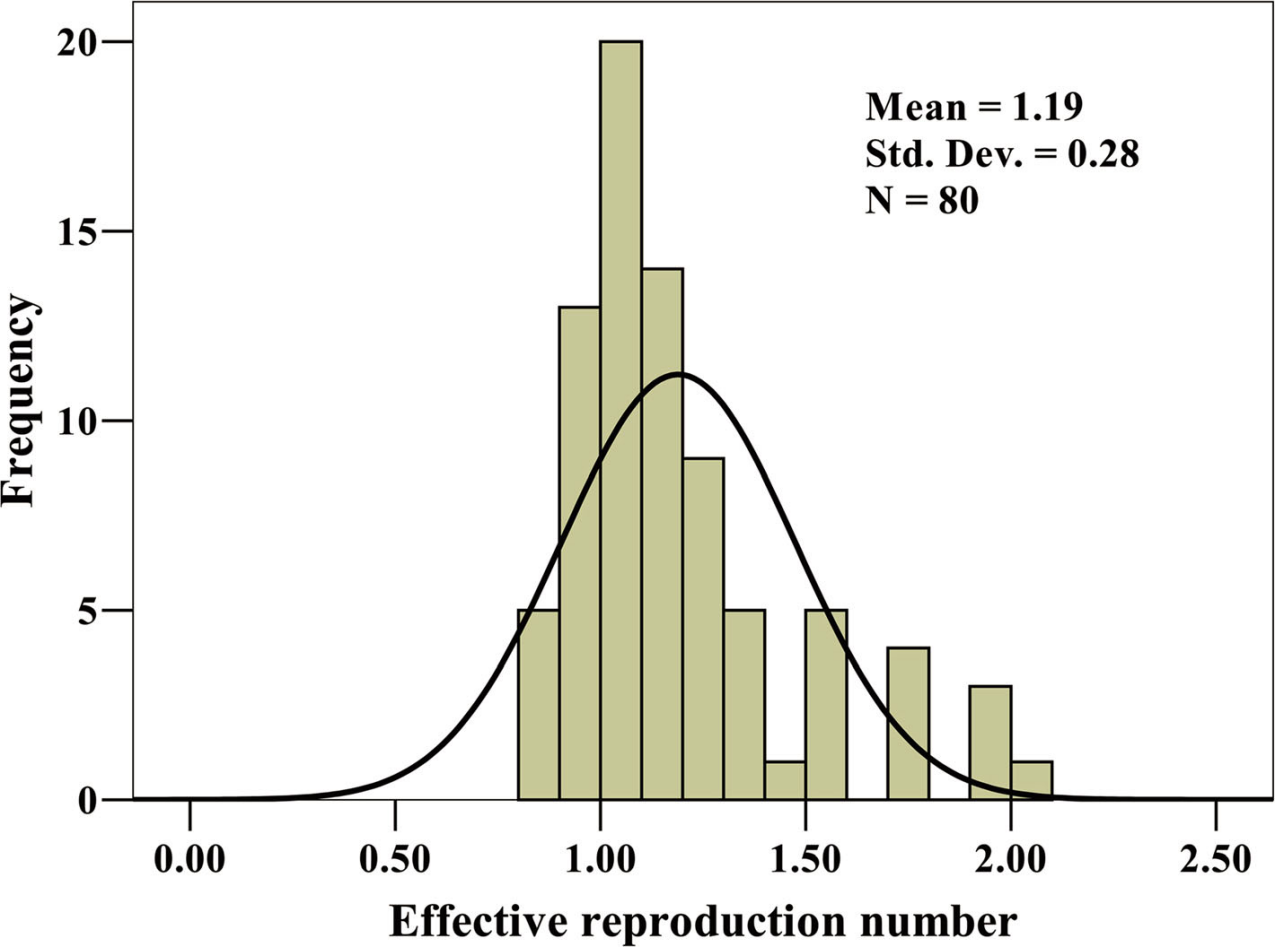
Distribution of *R*_*eff*_ estimated by Model 2 in Hubei Province, China

**Fig. 7:**
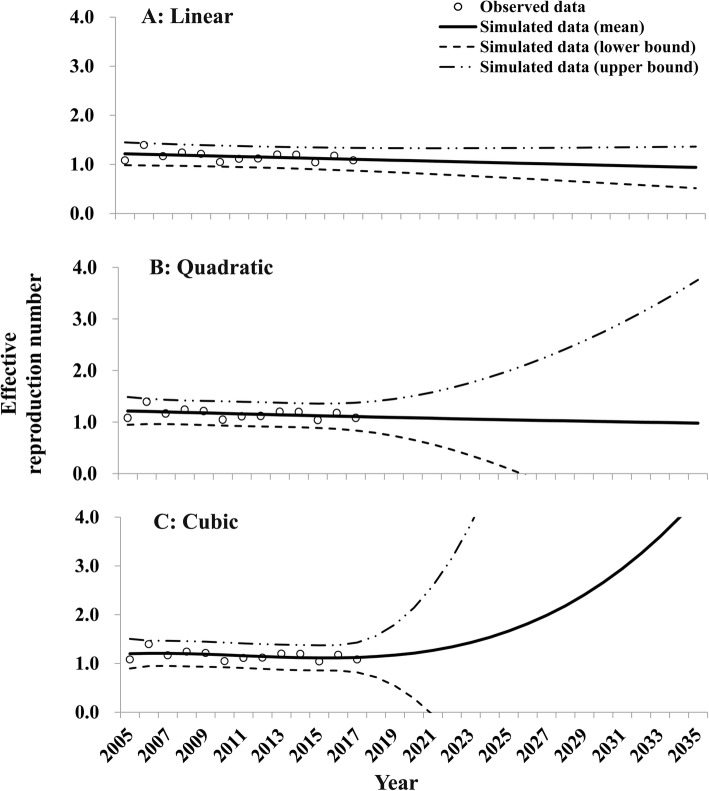
Simulated trend of *R*_*eff*_ in Hubei Province, China. **a**, Linear model; **b**, Quadratic model; **c**, Cubic model

The effective reproduction number *R*_*eff*_ of the disease was calculated with mean value of 1.19 (95% CI: 1.13–1.25) using Model 2 (Fig. 6). The mean value of *R*_*eff*_ was 1.08 (95% CI: 0.83–1.34) in the year of 2005. It reached a peak value of 1.39 (95% CI: 1.14–1.65) in 2006. Although it had a lowest value in 2010, it decreased slightly yearly (Table 4). By fitting the 11 equations with *R*_*eff*_ calculated by the SEIAR model with the reported data, the three most fitting models were Cubic, Linear, and Quadratic (Table 5). The fitted results were shown in Fig. 7. The Linear and Quadratic models forecasted that the mean value of *R*_*eff*_ would decrease yearly. Based on the Linear model, *R*_*eff*_ would reach a low value of 1.00 (95% CI: 0.67–1.34) in the year of 2028 and be down to an epidemic threshold of 0.99 (95% CI: 0.65–1.34) in 2029. Based on the Quadratic model, *R*_*eff*_ would reach a low value of 1.00 (95% CI: 0.00–2.93) in the year of 2031 and be down to an epidemic threshold of 0.999 (95% CI: 0.00–3.12) in 2032. However, the Cubic model predicted an increasing trend after the year 2018.

**Table 4:** The yearly *R*_*eff*_ estimated by Model 2 from 2005 to 2017 in Hubei Province, China

Year	Mean	Lower bound of 95% CI	Upper bound of 95% CI
2005	1.08	0.83	1.34
2006	1.39	1.14	1.65
2007	1.17	0.94	1.40
2008	1.24	1.00	1.48
2009	1.21	0.96	1.47
2010	1.05	0.75	1.34
2011	1.11	0.74	1.48
2012	1.12	0.92	1.31
2013	1.20	0.82	1.58
2014	1.20	0.98	1.42
2015	1.04	0.78	1.30
2016	1.18	0.74	1.62
2017	1.08	0.92	1.24

*CI* Confidence interval

**Table 5:** The fitting results of the 11 equations to the values of *R*_*eff*_ estimated by Model 2 from 2005 to 2017 in Hubei Province, China

Equation	Model summary		Parameter estimates			
	*R*^2^	*P*	Constant	b1	b2	b3
Linear	0.138	0.211	1.224	−0.009	–	–
Logarithmic	0.092	0.315	1.226	−0.038	–	–
Inverse	0.014	0.703	1.149	0.044	–	–
Quadratic	0.138	0.475	1.227	− 0.010	0.000	–
Cubic	0.151	0.671	1.183	0.021	−0.005	0.000
Compound	0.131	0.224	1.218	0.993	–	–
Power	0.083	0.339	1.219	−0.030	–	–
S	0.011	0.738	0.137	0.032	–	–
Growth	0.131	0.224	0.197	−0.007	–	–
Exponential	0.131	0.224	1.218	−0.007	–	–
Logistic	0.131	0.224	0.821	1.008	–	–

## Discussion

To our knowledge, this is the first study to estimate the long-term transmissibility and forecast the *R*_*eff*_ trend of Shigellosis in China. Located in the lake region, Hubei Province has a high burden of shigellosis and provides a large data for the modelling study. Our model developed in Hubei Province could also be used to study the transmission of the shigellosis in other places. Therefore, our study has an important significance to provide a reference for deeply understanding the transmission characteristics of the disease.

### Validity of the model

In our study, the SEIARW model was employed to fit the epidemic curves of the reported data, the results of Chi-square test showed a high good-of-fitness of our model to the reported data, suggesting that the model is suitable for this study and can be used to estimate the transmissibility of the disease.

### The epidemiological characteristics of the disease

In this study, we found that the incidence of the disease was still in a high level although with a decreasing trend. This finding is similar to the epidemiological characteristic in many areas in China [9–11, 18].

Our findings of sex and age distribution showed that: 1) male individuals were more likely to be infected by *Shigella spp* than females; 2) individuals who were infants or old were more likely to be infected by the pathogen. These findings are similar to the epidemiological characteristics of shigellosis [24, 32, 33]. The intervention focusing on male, children, newborns and elders might be more effective to prevent and control the disease. It is also important to monitor the disease among those populations.

Our findings of area distribution showed that the incidence of the disease had a high heterogeneity in different sub-regions of Hubei Province. Shigellosis had a high transmission in large cities such as Wuhan City (the capital city of the province) and Yichang City (which has a population more than 400 million) or cities near Wuhan City such as Xiantao City. We also found that the incidence of shigellosis in urban areas was higher than that in rural areas. It might due to the higher population density in urban areas. However, the transmissibility remains unclear in urban and rural areas and further research is needed to explore the force of the transmission in different areas.

About a half of the cases were diagnosed in 1.7 days from their illness onset date. However, this also meant that about 50% of the cases were diagnosed after 1.7 days from their illness onset date. There is a room to improve the surveillance system’s ability of diagnosing the disease immediately and to shorten the *D*_*ID*_ of Shigellosis cases.

### The transmissibility of shigellosis

The “knock-out” simulation showed that parameter *b*_*w*_ had almost no contribution during the transmission which meant that the transmission route of reservoir to person was interrupted in the area. This is mainly attributed to the government’s progress of improving sanitation drinking water and lavatories, food safety, and people’s health behaviors like drinking boiled water. Therefore, the remained major transmission route is person to person which will be the main public health focus to control the disease. This result also provided a mathematical evidence of the epidemiological characteristics of shigellosis that the pathogen was transmitted primarily through person-to-person [24]. Therefore, a combined strategy of case management would be strongly recommended including case isolation and treatment, environment disinfection, surveillance system improvement, and individuals’ health behaviors improvement like hand hygiene.

Our results showed that the mean value of *R*_*eff*_ of Shigellosis was 1.19, meaning that the expected number of secondary infections that resulted from introducing a single infected individual into an otherwise susceptible population was only 1.19 on average. This revealed that the transmissibility of shigellosis is lower than many other infectious diseases such as influenza, Ebola virus disease, and Norovirus infection [12, 14, 15, 34].

In addition, the transmissibility of Shigellosis decreased slightly from 2005 to 2017. Although the Linear, Quadratic, and Cubic models had the best goodness of fit, the 95%CIs of Quadratic model and Cubic model were not stable after the year 2019 (Fig. 7). Therefore, the Linear model was recommended to predict the trends of the transmissibility. The Linear model predicted that the *R*_*eff*_ would down to an epidemic threshold (*R*_*eff*_ = 1.00) in 2029. This was an interesting result which meant that the disease would probably be eliminated after the year of 2029. But this result was based on the assumption that the transmission condition would remain stable in the future years. Therefore, this trend might be different in other provinces. Hubei Province is located in a lake region with many cities suffered from floods frequently. The transmissibility of Shigellosis would be increased after the flood and provides a potential transmission route of water to person. On the other hand, the year of the transmission interrupted might be ahead of schedule since the disease control strategy would be strengthened in the future years, especially with the progress of “Health China” action launched by the Chinese government. More researches are needed to simulate the trend of transmissibility of the shigellosis with the interventions considered.

### Limitations

Limited by the availability of data, there are several limitations in this study.

Firstly, some environment factors (temperature, humidity, and rainfall) which might affect the spread of the disease were not considered in the model.

Secondly, due to the transmission heterogeneity, the features and trends of the transmissibility of the disease might be different in different areas in Hubei Province.

Thirdly, our study only focused in Hubei Province, the characteristics and the trends of transmissibility of shigellosis might be different in other provinces. The applicability of the mathematical models remains unclear to different datasets of reported shigellosis. However, Hubei Province has a high burden of shigellosis and provides us a large data for the modelling. The ordinary differential equation model has a strong applicability. It could be used in different populations including school or community with small-scale outbreaks [14, 19], and in different diseases such as shigellosis, norovirus infection, Ebola virus disease, and influenza [12, 14, 19, 31].

In addition, we did not simulate the sensitivity and specificity improvement of the surveillance system by the model. More researches are needed to perform the simulation of shorten *D*_*ID*_ in different scenarios.

## Conclusions

The disease burden of Shigellosis is still in a high level in Hubei Province, China. Individuals who were children, new-born, elders, and living in urban areas were more likely to be infected by *Shigella spp*. Most of the cases were diagnosed in 3 days from their illness onset date. The transmission route of reservoir to person had no contribution to the disease transmission in this area. The transmission of the disease would probably be interrupted in the year of 2029.

## Data Availability

The datasets used and analyzed during the current study are available from Dr. Qi Chen (chenqi8700@qq.com) on reasonable request.

## Abbreviations

CI: Confidence interval
*D*_*ID*_: Duration from illness onset date to diagnosed date
IQR: Inter-quartile range
SEIAR: Susceptible-exposed-symptomatic-asymptomatic-recovered
SEIARW: Susceptible-exposed-symptomatic-asymptomatic-recovered-water/food
